# T-cell differentiation and CD56+ levels in polypoidal choroidal vasculopathy and neovascular age-related macular degeneration

**DOI:** 10.18632/aging.101329

**Published:** 2017-11-20

**Authors:** Yousif Subhi, Marie Krogh Nielsen, Christopher Rue Molbech, Akio Oishi, Amardeep Singh, Mogens Holst Nissen, Torben Lykke Sørensen

**Affiliations:** ^1^ Clinical Eye Research Division, Department of Ophthalmology, Zealand University Hospital, Roskilde, Denmark; ^2^ Faculty of Health and Medical Science, University of Copenhagen, Copenhagen, Denmark; ^3^ Department of Ophthalmology and Visual Sciences, Kyoto University Graduate School of Medicine, Kyoto, Japan; ^4^ Department of Ophthalmology, Skåne University Hospital Malmö-Lund, Lund, Sweden; ^5^ Eye Research Unit, Department of Immunology and Microbiology, University of Copenhagen, Copenhagen, Denmark

**Keywords:** polypoidal choroidal vasculopathy, age-related macular degeneration, T-cells, immunosenescence

## Abstract

Polypoidal choroidal vasculopathy (PCV) and neovascular age-related macular degeneration (AMD) are prevalent age-related diseases characterized by exudative changes in the macula. Although they share anatomical and clinical similarities, they are also distinctly characterized by their own features, e.g. vascular abnormalities in PCV and drusen-mediated progression in neovascular AMD. PCV remains etiologically uncharacterized, and ongoing discussion is whether PCV and neovascular AMD share the same etiology or constitute two substantially different diseases. In this study, we investigated T-cell differentiation and aging profile in human patients with PCV, patients with neovascular AMD, and age-matched healthy control individuals. Fresh venous blood was prepared for flow cytometry to investigate CD4^+^ and CD8^+^ T-cell differentiation (naïve, central memory, effector memory, effector memory CD45ra^+^), loss of differentiation markers CD27 and CD28, and expression of aging marker CD56. Patients with PCV were similar to the healthy controls in all aspects. In patients with neovascular AMD we found significantly accelerated T-cell differentiation (more CD28^−^CD27^−^ cells) and aging (more CD56^+^ cells) in the CD8^+^ T-cell compartment. These findings suggest that PCV and neovascular AMD are etiologically different in terms of T cell immunity, and that neovascular AMD is associated with T-cell immunosenescence.

## INTRODUCTION

Aging is the greatest risk factor of developing age-related macular degeneration (AMD) — the most common reason of irreversible vision loss and blindness in the elderly [1,2]. The late stage of the disease is characterized by choroidal neovascularizations (CNV) which protrude through the basal membrane (Bruch's membrane) of retinal pigment epithelium (RPE) and into the neuroretina leading to visual symptoms such as metamorphopsia, blurred vision, and scotoma [3]. This stage of the disease is called neovascular AMD and is associated with retinal aging and dysfunction [5]. Alterations of the innate and adaptive immune system in the elderly play a key role in retinal immune homeo-stasis, vascular endothelial growth factor (VEGF) expression upon injury, and promotion of angiogenesis [6–11]. Polypoidal choroidal vasculopathy (PCV) is an important differential diagnosis to neovascular AMD with shared visual symptoms but different anatomical and clinical characteristics [12,13]. PCV is charac-terized by the acquired polypoidal deformations at the terminal of CNVs [12,13]. These polyps protrude into the sub-RPE space, often with accompanying branching vascular networks [12,13]. Differences with neovascular AMD may seem subtle, but diagnosing PCV have important clinical implications: neovascular AMD is managed solely by regular intravitreal injections with VEGF inhibitors, whereas PCV may benefit from a combination of VEGF inhibitors to limit exudates and photodynamic therapy for polyp regression [12–16]. PCV is diagnosed in ~6-10 % of Caucasians and ~50 % of Asians with presumed neovascular AMD who undergo detailed retinal diagnosis using indocyanine green retinal angiography [12,13,17]. Hence, PCV is a relatively common disease with distinct clinical and anatomical differences [18], but very little is known regarding its etiology. One important question remains unanswered: is PCV a different clinical manifestation of a disease that etio-logically is the same as neovascular AMD, or should we consider PCV a disease that is distinguished by its own etiology?

Comparative etiological studies of PCV and neovascular AMD are sparse. Tong et al. found that intraocular levels of VEGF is increased in eyes with PCV, but at a lower level than in eyes with neovascular AMD [19]. Zeng et al. investigated extracellular tissue homeostasis and found increased level of serum matrix metalloproteinases 2 and 9 in patients with PCV compared to patients with neovascular AMD and healthy controls [20]. Genetic studies suggest both similarities and differences and at present generally neither confirm nor reject a possible association of PCV with alterations in the immune system [21].

Immune dysfunction plays a key role in the etiology of neovascular AMD [22]. Important findings include complement dysfunction [23,24], altered innate immune system [6–8,22], and changes in the adaptive immune system [6,9–11]. The latter is an emerging field of research with interesting findings so far suggesting that the adaptive immune system may be involved in the etiology of neovascular AMD. We previously found that patients with neovascular AMD have a lower proportion of T helper 1 cells as well as dysfunctions in the expression of T-cell chemokine receptors [9,10]. Murine models of experimental CNV find systemic activation of T helper cells [25]. In vitro studies of activated T-cells and their cytokines have demonstrated two important mediators in the etiology by modulating the protein expression and secretion in retinal pigment epithelium (RPE) cells: increased expression and secretion of chemokines [26] and increased expression and secretion of complement proteins [27] that constitute a main part of retinal drusen formation — the *sine qua non* of AMD.

Considering that PCV and neovascular AMD are only seen in the aged, we turned our attention to immuno-senescence — age-related changes of the immune system [28]. The thymic output of T-cells peaks at puberty and declines gradually afterwards, and the declined running supply of naïve T-cells consequently results in a higher ratio of more differentiated T-cells [28]. Differentiated and activated T-cells become central memory or effector memory T-cells with different set of surface markers and function [29]. T-cell differentiation and proliferation also leads to gradual loss of CD27 and CD28 expression: CD4^+^ T-cells lose CD27 first and CD28 later; whereas the opposite is the case for CD8^+^ T-cells, which lose CD28 first, and CD27 later [29]. Details on T-cell differentiation profile are not previously investigated in patients with PCV or neovascular AMD. We previously investigated CD56 expression on CD28^−^ T-cells and found significant differences between patients with AMD and healthy controls [11]. CD56 is a surface marker of natural killer cells, but is also expressed broadly among leukocyte subsets [30]. In T-cells, CD56 expression is linked to an increased cytolytic activity [30]. However, from immunosenescence point-of-view, CD56 is interesting since it is one of the best described markers of T-cell aging [31–33]. CD56 expression has not been studied in patients with PCV and the role of T-cells in PCV remains unexplored.

Our aim with this study was to investigate T-cell aging and differentiation by mapping the differentiation profile and investigating the proportion of CD56^+^ T-cells in different differentiation subsets in patients with PCV and compare the results to that of patients with neovascular AMD and healthy controls.

## RESULTS

We recruited 24 patients with PCV, 50 patients with neovascular AMD, and 26 healthy controls. We post-hoc excluded five patients with neovascular AMD and two healthy controls because we suspected an ongoing acute immune response due to elevated plasma C-reactive protein levels (> 15 mg/L). Therefore, our analyses are based on 24 patients with PCV, 45 patients with neovascular AMD, and 24 healthy controls. Participant characteristics (demographics, co-morbidi-ties, and lifestyle factors) did not differ significantly between the groups (Table 1).

**Table 1:** Detailed participant characteristics

	Patients with PCV(n=24)	Patients with nAMD (n=47)	Healthy controls(n=24)	*P*-value
**Demographics**				
Age, years, mean (SD)	72.5 (7.9)	75.8 (7.3)	73.4 (7.7)	0.20 ^a^
Females, n, (%)	15 (63)	23 (51)	15 (63)	0.54 ^b^
**Co-morbidities**				
Hypertension, n (%)	9 (38)	23 (51)	7 (29)	0.19 ^b^
Cardiovascular disease, n (%)	4 (17)	10 (22)	2 (8)	0.38 ^c^
Hypercholesterolemia, n (%)	7 (29)	10 (22)	6 (25)	0.82 ^b^
Type 2 diabetes, n (%)	2 (8)	6 (13)	0 (0)	0.17 ^c^
**Lifestyle factors**				
Smoking, n (%)				0.091 ^c^
Current	8 (33)	14 (31)	3 (12)	
Previous	13 (54)	18 (40)	10 (42)	
Never	3 (13)	13 (29)	11 (46)	
Alcohol consumption, units, median (IQR)	4 (1 to 12)	3 (1 to 9)	4 (2 to 7)	0.67 ^d^
Body mass index, mean (SD)	24.4 (3.4)	26.2 (4.0)	25.7 (3.1)	0.16 ^a^
Physically active, n (%)	13 (54)	23 (51)	17 (71)	0.27 ^b^

Abbreviations: PCV = polypoidal choroidal vasculopathy; nAMD = neovascular age-related macular degeneration; SD = standard deviation; IQR = interquartile range.

Statistical comparisons are made using (a) one-way analysis of variance, (b) Χ^2^-test, (c) Fisher's Exact test because of categories with ≤ 4 cases, and (d) Kruskal-Wallis' test.

### Counts and percentages of CD4^+^ and CD8^+^ T-cells

**Figure 1:**
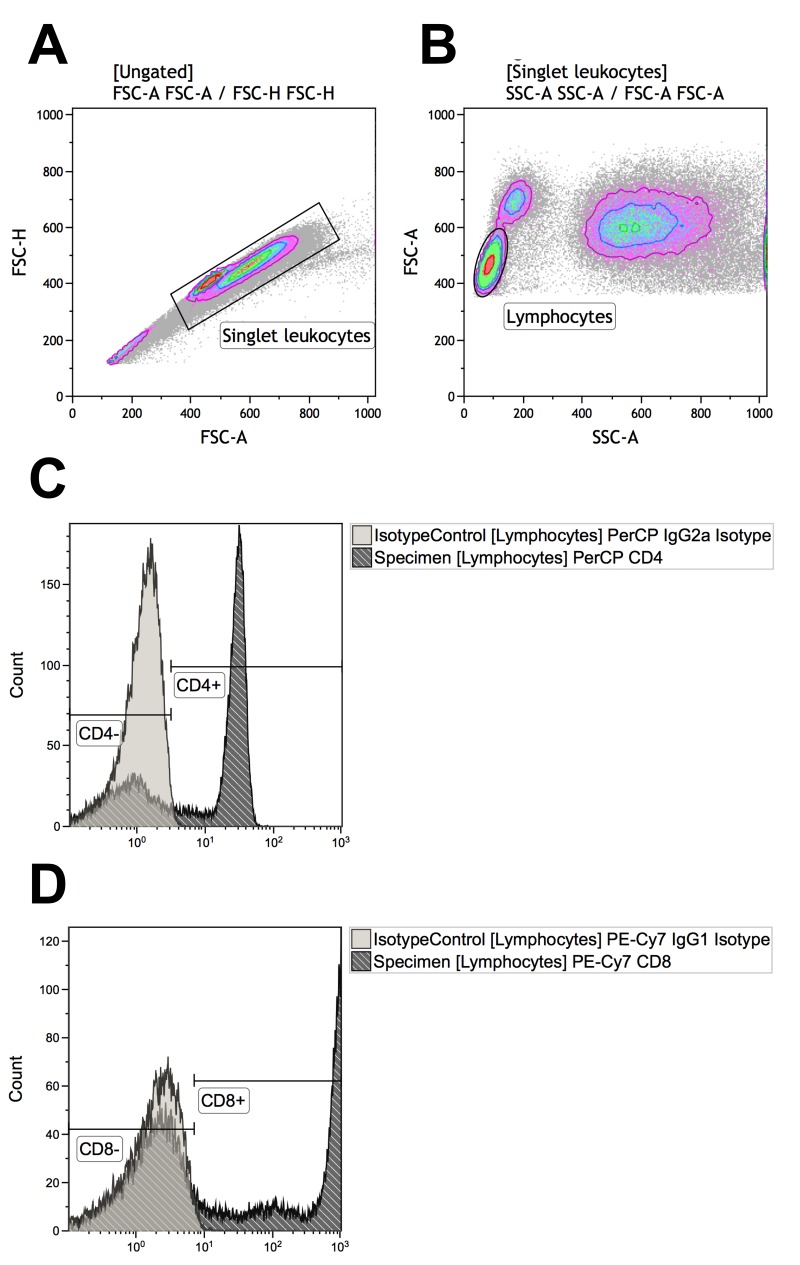
Gating strategies for identifying T-cells that are CD4^+^ (defined as CD4^+^CD8^−^) or CD8^+^ (defined as CD4^−^CD8^+^) (**A**) Singlet leukocytes were identified using forward scatter height vs. area scatter on a combined contour and density plot. (**B**) Lymphocytes were identified using forward scatter area vs. side scatter area on a combined contour and density plot. (**C**) CD4^+^ and CD4^−^ lymphocytes were identified with the help of negative isotype controls (set at 1% to distinguish fluorescence signal from non-specific background signals). (**D**) CD4^+^ and CD4^−^ lymphocytes were identified with the help of negative isotype controls (set at 1% to distinguish fluorescence signal from non-specific background signals). Using logical Boolean sequences, we distinguished two T-cell populations for further analyses: CD4^+^ T-cells that were CD4^+^CD8^−^ and CD8^+^ T-cells that were CD4^−^CD8^+^.

We first identified CD4^+^ and CD8^+^ T-cells (Figure 1). Groups did not differ significantly in CD4^+^ and CD8^+^ T-cells counts and percentages. Patients with PCV had a mean CD4^+^ T-cell count of 846 (SD: 414) cells/mm^3^ constituting 43 (SD: 13) % of total lymphocytes, not significantly different from that in patients with neovascular AMD (count: mean 754 (SD: 334) cells/mm^3^; percentage: mean 45 (SD: 12) %) or healthy controls (count: mean 796 (SD: 204) cells/mm^3^, percentage: 48 (SD: 9) %) (P = 0.55 and P = 0.37, for count and percentage respectively, using one-way analysis of variance). Patients with PCV had a mean CD8^+^ T-cell count of 581 (SD: 224) cells/mm^3^ constituting 31 (SD: 10) % of total lymphocytes, also not significantly different from that in patients with neovascular AMD (count: mean 499 (SD: 197) cells/mm^3^; percentage: mean 31 (SD: 10) %) or healthy controls (count: mean 504 (SD: 261) cells/mm^3^, percentage: 29 (SD: 10) %) (P = 0.32 and P = 0.73, for count and percentage respectively, using one-way analysis of variance).

### Differentiation profile of CD4^+^ and CD8^+^ T-cells

**Figure 2:**
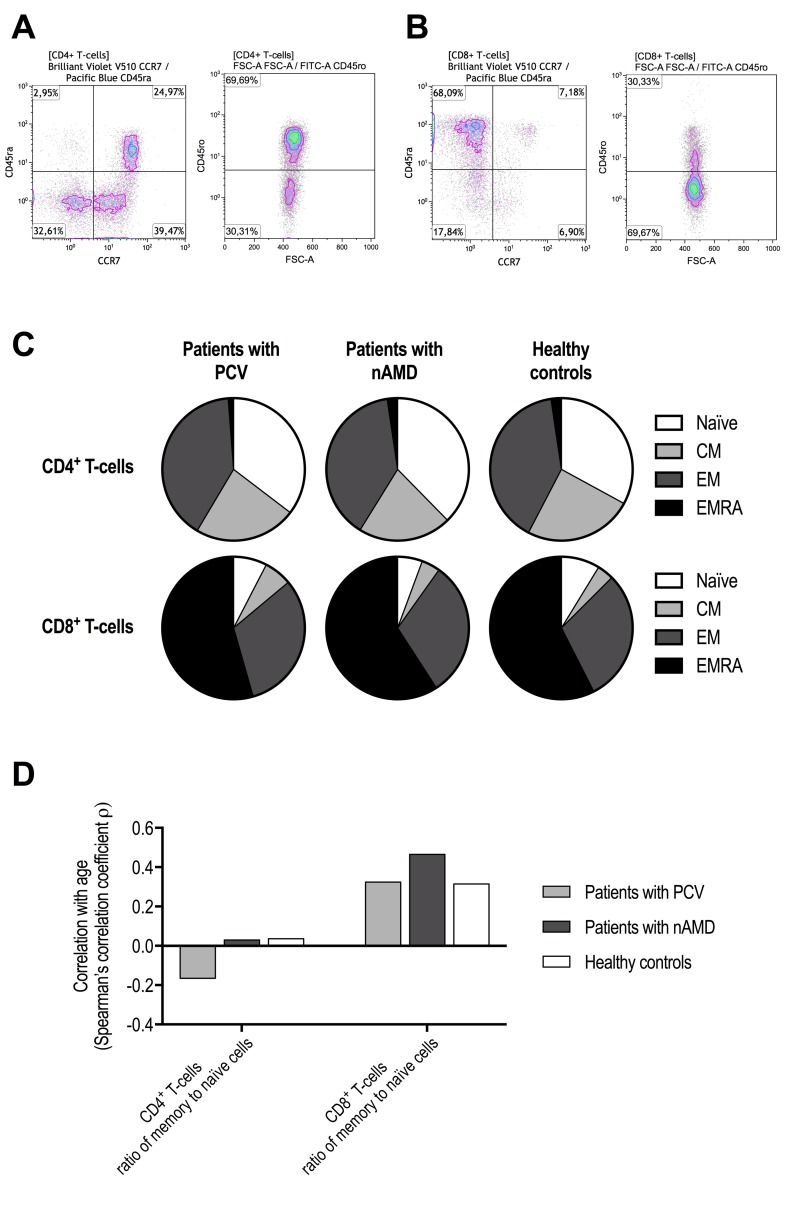
Functional differentiation profile of CD4^+^ and CD8^+^ T-cells in patients with polypoidal choroidal vasculopathy (PCV), patients with neovascular age-related macular degeneration (nAMD), and healthy controls Cells were defined as naïve T-cells CD45ra^+^CD45ro^−^CCR7^+^, central memory T-cells (CM) CD45ra^−^CD45ro^+^CCR7^+^, effector memory T-cells (EM) CD45ra^−^CD45ro^+^CCR7^−^, and effector memory CD45ra^+^ T-cells (EMRA) CD45ra^+^CD45ro^+^CCR7^−^. These subsets were separately identified in CD4^+^ T-cells (**A**) and CD8^+^ T-cells (**B**). (**C**) We found no significant differences between patients with PCV, patients with nAMD, and healthy controls (P > 0.05 for all comparisons using Kruskal-Wallis test). (**D**) Age-related changes in the rat**i**o of memory to naïve T-cells indicated no age-related change in CD4^+^ T-cells, but a clear age-related increase in CD8^+^ T-cells that appeared stronger in patients with nAMD.

The functional differentiation profile (naïve T-cells, central memory T-cells, effector memory T-cells, effector memory CD45ra^+^ T-cells) in CD4^+^ and CD8^+^ T-cells did not differ significantly between patients with PCV, patients with neovascular AMD, and healthy controls (P > 0.05 for all comparisons using Kruskal-Wallis test) (Figure 2). We found no significant differences between groups in memory to naïve T-cell ratio in CD4^+^ T-cells (P = 0.95, Kruskal-Wallis test) or in CD8^+^ T-cells (P = 0.15, Kruskal-Wallis test). Memory to naïve T-cell ratio in CD4^+^ T-cells was not correlated with age in patients with PCV, patients with neovascular AMD, or in healthy controls; however, in CD8^+^ T-cells we observed a weak correlation in patients with PCV (ρ = +0.3, Spearman's correlation) and in healthy controls (ρ = +0.3, Spearman's correlation), which was stronger in patients with neovascular AMD (ρ = +0.5, Spearman's correlation) (Figure 2).

### Loss of CD27 and CD28 expression in CD4^+^ and CD8^+^ T-cells

**Figure 3:**
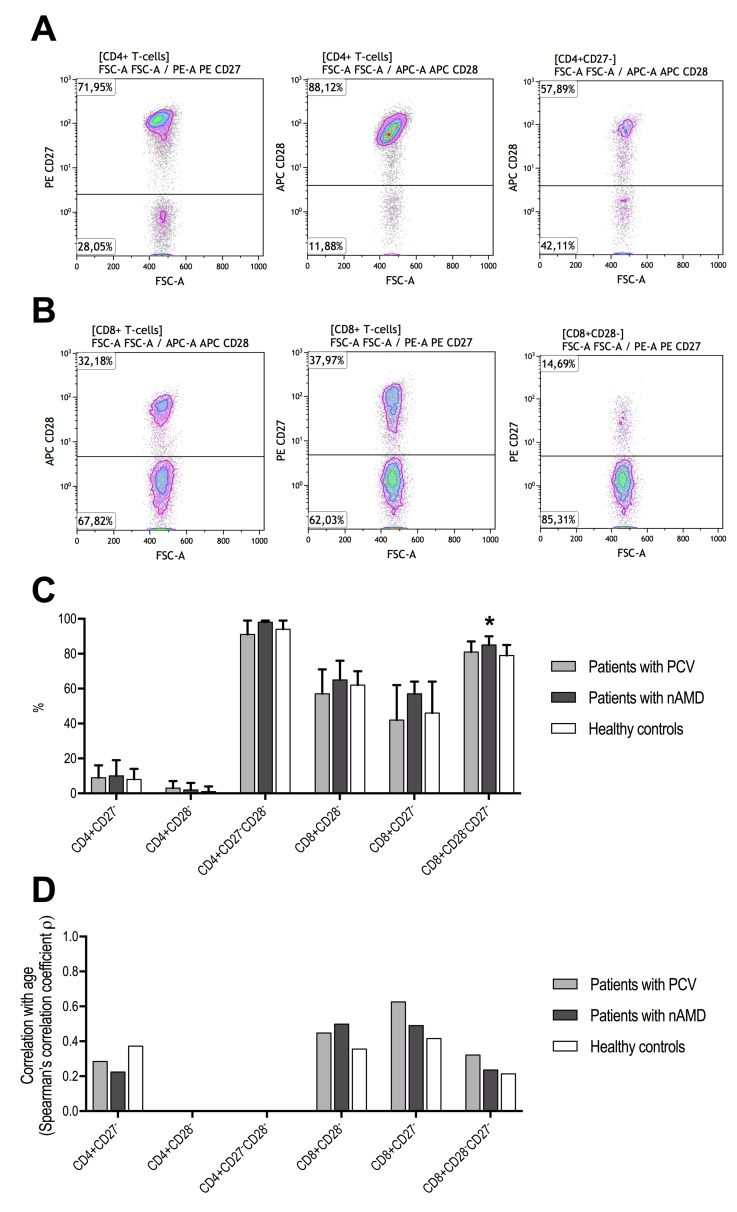
T-cell differentiation measured as CD27 and CD28 downregulation in patients with polypoidal choroidal vasculopathy (PCV), patients with neovascular age-related macular degeneration (nAMD), and healthy controls CD4^+^ T-cells lose CD27 first and CD28 later, whereas the opposite is the case for CD8^+^ T-cells which lose CD28 first and CD27 later. (**A**) Identification of CD27^−^, CD28^−^, and CD28^−^ in CD27^−^ CD4^+^ T-cells. (**B**) Identification of CD28^−^, CD27^−^, and CD27^−^ in CD28^−^ CD8^+^ T-cells. (**C**) We only found a difference in CD27^−^ in CD28^−^ CD8^+^ T-cells (signified with *, P = 0.040, Kruskal-Wallis test), where patients with nAMD which had significantly higher proportion compared to the other groups. (**D**) Age-related changes in CD27 and CD28 loss were comparable between the groups. CD4+CD28- and CD4+CD27-CD28- were not included due to numbers close to the extremes yielding unstable correlation analyses.

Loss of CD27 and CD28 expression in T-cells were correlated to the functional differentiation profile as expected (Table 2). Differences in T-cell differentiation between groups were observed when comparing loss of CD27 and CD28. Loss of CD27 and CD28 expression in CD4^+^ T-cells did not differ patients with PCV and patients with neovascular AMD and were comparable to healthy controls (Figure 3). Increasing age was weakly correlated with loss of CD27 expression in CD4^+^ T-cells and not subject to group differences (Figure 3). In patients with neovascular AMD, we observed that loss of CD27 in CD8^+^ CD28^−^ T-cells was significantly increased compared to healthy controls (P = 0.019, Mann-Whitney U-test), whereas no significant differences were observed between patients with PCV and healthy controls (P = 0.56, Mann-Whitney U-test) (Figure 3). Loss of CD28 and CD27 in CD8^+^ T-cells were moderately correlated with age in patients with PCV (CD28^−^ : ρ = +0.6, Spearman's correlation; CD27^−^ : ρ = +0.4, Spearman's correlation), patients with neo-vascular AMD (CD28^−^ : ρ = +0.5, Spearman's correlation; CD27^−^ : ρ = +0.5, Spearman's correlation), and in healthy controls (CD28^−^ : ρ = +0.4, Spearman's correlation; CD27^−^ : ρ = +0.4, Spearman's correlation). Similar results were observed in CD8^+^CD28^−^CD27^−^ but with more attenuated correlation coefficients (Figure 3).

**Table 2:** Loss of CD27 and CD28 expression on CD4^+^ and CD8^+^ T-cells subsets of functional differentiation

	Naïve	CM	EM	EMRA
**CD4^+^ T-cells**				
% CD27^−^, median (IQR)	0 (0 to 0)	1 (1 to 2)	21 (13 to 29)	12 (5 to 46)
% CD28^−^, median (IQR)	0 (0 to 0)	0 (0 to 0)	2 (0 to 9)	8 (2 to 37)
% CD28^−^ in CD27^−^ cells, median (IQR)	*Very few cells*	*Very few cells*	10 (1 to 36)	64 (17 to 87)
**CD8^+^ T-cells**				
% CD28^−^, median (IQR)	5 (2 to 12)	1 (0 to 2)	24 (11 to 42)	92 (88 to 95)
% CD27^−^, median (IQR)	3 (1 to 7)	2 (1 to 4)	18 (11 to 29)	80 (72 to 87)
% CD27^−^ in CD28^−^ cells, median (IQR)	*Very few cells*	*Very few cells*	55 (33 to 73)	87 (79 to 92)

Abbreviations: CM = central memory; EM = effector memory; EMRA = effector memory CD45ra positive; IQR = interquartile range.

CD27 and CD28 downregulation reflect T-cell differentiation level. CD4+ T-cells lose CD27 first and CD28 later, whereas the opposite is the case for CD8+ T-cells which lose CD28 first and CD27 later. These changes are also reflected when looked in CD4+ and CD8+ T-cell subsets of functional differentiation.

### Percentage of CD56^+^ cells in CD4^+^ and CD8^+^ T-cells

**Figure 4:**
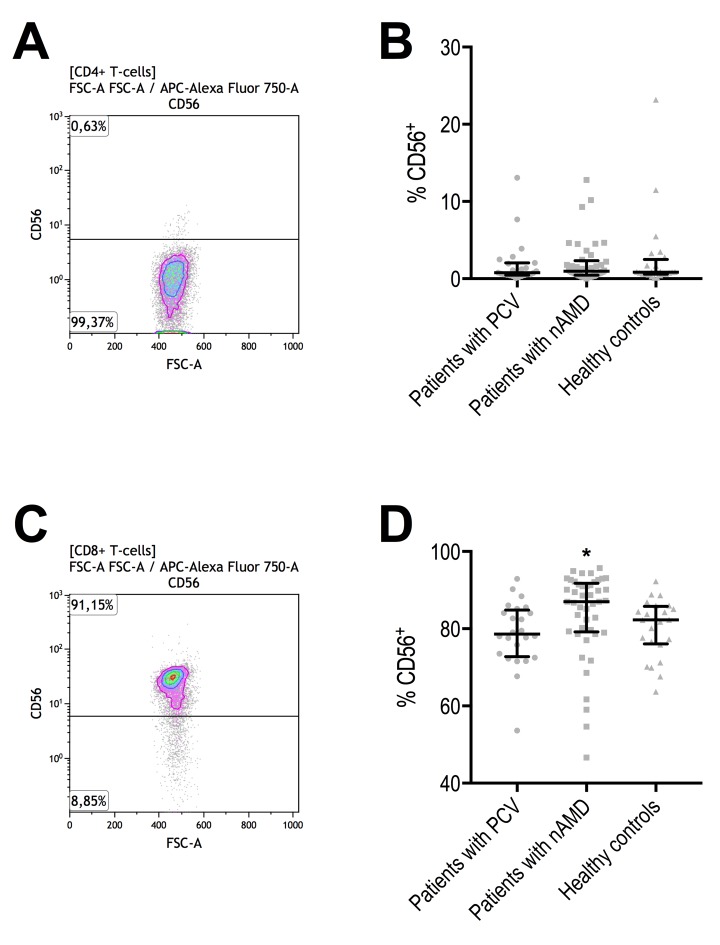
Proportion of CD56^+^ CD4^+^ and CD8^+^ T-cells in patients with polypoidal choroidal vasculopathy (PCV), patients with neovascular age-related macular degeneration (nAMD), and healthy controls (**A**) CD56^+^ CD4^+^ T-cells were identified with the help of negative isotype controls (set at 1% to distinguish fluorescence signal from non-specific background signals). (**B**) CD56+ in CD4+ T-cells did not differ between the groups. Horizontal line with whiskers indicate median and interquartile range. (**C**) CD56^+^ CD8^+^ T-cells were identified with the help of negative isotype controls (set at 1% to distinguish fluorescence signal from non-specific background signals). (**D**) CD56+ in CD8+ T-cells differed significantly and was significantly higher in patients with nAMD (signified with *, P = 0.0016, Kruskal-Wallis test).

**Figure 5:**
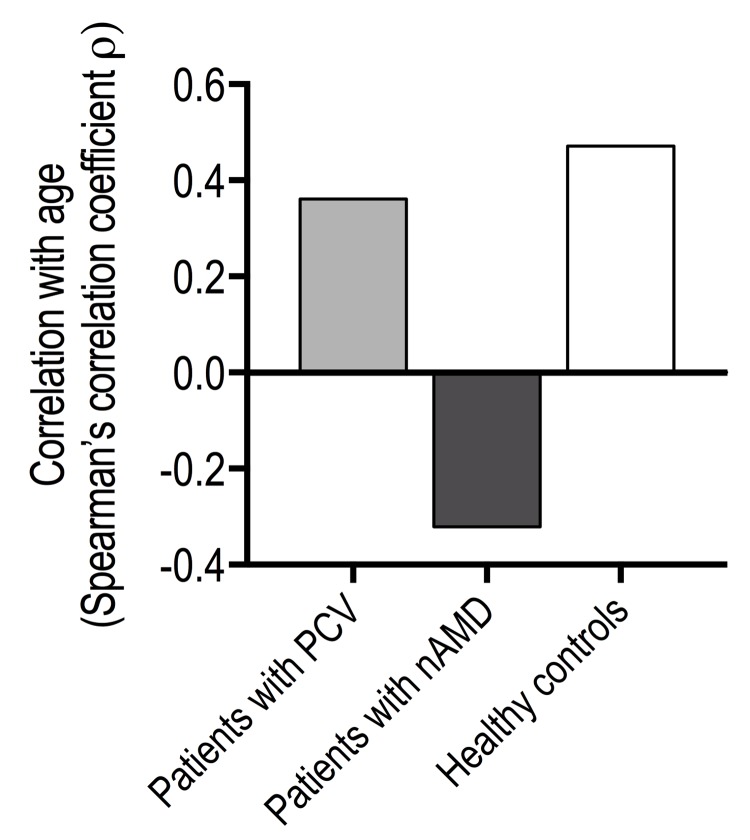
Age-related changes in % of CD56^+^ CD8^+^ T-cells in patients with polypoidal choroidal vasculopathy (PCV), patients with neovascular age-related macular degeneration (nAMD), and healthy controls

Patients with PCV did not differ from patients with neovascular AMD in CD56^+^ cells in CD4^+^ T-cells, and both patient groups had levels similar to that in healthy controls (Figure 4). However, while CD56^+^ in CD8^+^ T-cells were comparable between patients with PCV and healthy controls, it was significantly increased in patients with neovascular AMD (Figure 4). Aging correlated moderately with CD56 surface expression in CD8^+^ T-cells in patients with PCV (ρ = +0.4, Spearman's correlation) and in healthy controls (ρ = +0.5, Spearman's correlation). In patients with neo-vascular AMD, we observed an inverse correlation between age and CD56^+^ CD8^+^ T-cells (ρ = -0.3, Spearman's correlation) (Figure 5). Because of this finding and to further investigate aging and CD56^+^ cells in CD8^+^ T-cells, we stratified all participants on age and repeated the comparisons across groups of diagnosis (Table 3). Patients with PCV and patients with neovascular AMD had levels comparable to healthy controls among those aged >70 and ≤80 years and those aged >80 years. However, among the younger participants (aged ≤70 years), patients with neovascular AMD had significantly higher CD56^+^ expression in CD8^+^ T-cells than patients with PCV (P = 3.2 × 10^−4^, Mann-Whitney U test) or healthy controls (P = 9.1 × 10^−5^, Mann-Whitney U test) (Table 3).

**Table 3:** Aging marker CD56 expression on CD8^+^ T-cells in different age-ranges in patients with polypoidal choroidal vasculopathy (PCV), patients with neovascular age-related macular degeneration (nAMD), and healthy controls

	Patients with PCV (n = 24)	Patients with nAMD(n = 45)	Healthy controls(n = 24)	*P*-value
**Aged ≤70 years**	**(n = 10)**	**(n = 11)**	**(n = 8)**	
% CD56^+^, median (IQR)	75 (72 to 78)	**91 (87 to 93)**	74 (70 to 80)	**0.00030**
**Aged >70 and ≤80 years**	**(n = 10)**	**(n = 23)**	**(n = 11)**	
% CD56^+^, median (IQR)	83 (78 to 86)	86 (79 to 91)	83 (80 to 87)	0.48
**Aged >80 years**	**(n = 4)**	**(n = 13)**	**(n = 5)**	
% CD56^+^, median (IQR)	81 (76 to 85)	84 (76 to 90)	84 (84 to 86)	0.86

Abbreviations: PCV = polypoidal choroidal vasculopathy; nAMD = neovascular age-related macular degeneration; IQR = interquartile range.

Statistical comparisons are made using Kruskal-Wallis' test. Bold text indicates a statistically significant difference.

### Percentage of CD56^+^ cells by CD4^+^ and CD8^+^ T-cell differentiation level

We did not find any differences in CD56^+^ cells in any functional differentiation subset of CD4^+^ T-cells (Table 4). CD56 expression in Naïve and EMRA CD8^+^ T-cells also did not differ between the groups (P = 0.11 and P = 0.092, for Naïve and EMRA CD8^+^ T-cells, respectively, using Kruskal-Wallis test) (Table 4). Patients with neovascular AMD has significantly more CD56^+^ in EM cells compared to healthy controls (P = 0.0065, Mann-Whitney U test), whereas CM was not significantly different between the two groups (P = 0.23, Mann-Whitney U test). Patients with neovascular AMD had significantly more CD56^+^ in both CM (P = 0.0063, Mann-Whitney U test) and EM (P = 0.031, Mann-Whitney U test) when compared to patients with PCV. CD56^+^ in CM and EM in CD8^+^ T-cells did not differ significantly between patients with PCV and healthy controls (P = 0.28 and P = 0.77, for CM and EM, respectively, using Mann-Whitney U test).

**Table 4:** Aging marker CD56 expression in CD4^+^ and CD8^+^ T-cells of functional differentiation profile in relation to patients with polypoidal choroidal vasculopathy (PCV), patients with neovascular age-related macular degeneration (nAMD), and healthy controls

	Patients with PCV (n = 24)	Patients with nAMD (n = 45)	Healthy controls(n = 24)	*P*-value
**CD4^+^ T-cells**				
% Naïve CD56^+^, median (IQR)	0 (0 to 0)	0 (0 to 0)	0 (0 to 0)	0.55
% CM CD56^+^, median (IQR)	0 (0 to 1)	1 (0 to 2)	1 (0 to 2)	0.20
% EM CD56^+^, median (IQR)	0 (0 to 1)	0 (0 to 1)	1 (0 to 1)	0.28
% EMRA CD56^+^, median (IQR)	2 (0 to 19)	1 (0 to 10)	1 (0 to 6)	0.43
**CD8^+^ T-cells**				
% Naïve CD56^+^, median (IQR)	92 (82 to 96)	95 (90 to 97)	94 (88 to 96)	0.11
% CM CD56^+^, median (IQR)	92 (87 to 96)	**96 (94 to 98)**	95 (89 to 98)	**0.027**
% EM CD56^+^, median (IQR)	88 (81 to 94)	**93 (89 to 96)**	88 (83 to 91)	**0.011**
% EMRA CD56^+^, median (IQR)	70 (64 to 81)	79 (67 to 88)	75 (62 to 81)	0.092

Abbreviations: PCV = polypoidal choroidal vasculopathy; nAMD = neovascular age-related macular degeneration; IQR = interquartile range; CM = central memory; EM = effector memory; EMRA = effector memory CD45ra^+^.

Naïve T-cells are CD45ra+CD45ro-CCR7+, CM T-cells are CD45ra-CD45ro+CCR7+, EM T-cells are CD45ra-CD45ro+CCR7-, and EMRA T-cells are CD45ra+CD45ro+CCR7-.

Statistical comparisons are made using Kruskal-Wallis' test. Bold text indicates a statistically significant difference.

CD56^+^ in CD27^−^ and CD28^−^ cells did not differ significantly between groups in CD4^+^ T-cells (Table 5). In CD8^+^ T-cells, significantly more CD56^+^ was observed in CD28^−^, CD27^−^, and CD28^−^CD27^−^ subsets in patients with neovascular AMD when compared to patients with PCV (P = 0.0028, P = 0.022, P = 0.0070, CD28^−^, CD27^−^, and CD28^−^CD27^−^ respectively, using Mann-Whitney U test) or healthy controls (P = 0.048, P = 0.044, P = 0.062, CD28^−^, CD27^−^, and CD28^−^CD27^−^ respectively, using Mann-Whitney U test) (Table 5). CD56^+^ in CD28^−^ (P = 0.67, Mann-Whitney U test), CD27^−^ (P = 0.21, Mann-Whitney U test), and CD28^−^CD27^−^ (P = 0.33, Mann-Whitney U test) did not differ significantly between patients with PCV and healthy controls.

**Table 5:** Aging marker CD56 expression in CD27^−^ and CD28^−^ cells in patients with polypoidal choroidal vasculopathy (PCV), patients with neovascular age-related macular degeneration (nAMD), and healthy controls

	Patients with PCV(n = 24)	Patients with nAMD(n = 45)	Healthy controls(n = 24)	*P*-value
**CD4^+^ T-cells**				
% CD27^−^CD56^+^, median (IQR)	3 (1 to 12)	3 (1 to 12)	2 (1 to 10)	0.93
% CD28^−^CD56^+^, median (IQR)	7 (1 to 28)	4 (0 to 22)	7 (0 to 24)	0.94
% CD27^−^CD28^−^CD56^+^, median (IQR)	7 (0 to 27)	8 (0 to 32)	10 (0 to 27)	0.97
**CD8^+^ T-cells**				
% CD28^−^CD56^+^, median (IQR)	73 (64 to 81)	**84 (69 to 88)**	76 (63 to 83)	**0.030**
% CD27^−^CD56^+^, median (IQR)	72 (60 to 81)	**84 (72 to 88)**	79 (65 to 83)	**0.0057**
% CD28^−^CD27^−^CD56^+^, median (IQR)	70 (58 to 81)	**84 (68 to 88)**	77 (64 to 83)	**0.014**

Abbreviations: PCV = polypoidal choroidal vasculopathy; nAMD = neovascular age-related macular degeneration; IQR = interquartile range. Statistical comparisons are made using Kruskal-Wallis' test. Bold text indicates a statistically significant difference.

## DISCUSSION

Are PCV and neovascular AMD etiologically similar or different? In this study, we looked at this question in terms of systemic T-cell profile of differentiation and aging. Patients with PCV were similar to healthy controls in all aspects of T-cells differentiation and percentage of CD56^+^ T-cells. In contrast, we found multiple sources of evidence suggesting a more accele-rated differentiation and aging profile in patients with neovascular AMD. Ample evidence links an aging immune system to the presence of nonspecific inflam-mation, which accelerate development of age-related degenerative diseases. Our findings are in line with the general conception of neovascular AMD as a degene-rative disease of the macula and within that framework, immunosenescence as a contributing or an accompany-ing factor may not come as a surprise. Neurodegene-ration and inflammation lead to drusen formation in AMD which precedes formation of the new choroidal vessels that defines neovascular AMD. The lack of an association with drusen formation in PCV have so far raised the question of whether or not AMD and PCV may differ etiologically [34]. Based on our findings in this study, we suggest that PCV should be considered etiologically different from neovascular AMD, at least in terms of T-cell differentiation and aging.

The T-cell pool in elderly humans are maintained by proliferation of the existing naïve T-cells [35,36]. Naïve T-cells are eventually spent due to antigen exposure and differentiate into CM, and eventually into EM and EMRA, also known as terminal effector memory cells. Näive T-cells are characterized as being highly proliferative and lightly cytotoxic T-cells in contrast to memory cells which are less proliferative but highly cytotoxic [37]. Previous studies on aging and T-cells have demonstrated that in numbers, naïve CD4+ T-cells remain relatively stable with age [36,38], whereas naïve CD8+ T-cells undergo a substantial age-related decrement [36,38,39]. This is in line with our findings where the memory-to-naïve T-cell ratio remained relatively unchanged with age in CD4^+^ T-cells and increased with age in CD8^+^ T-cells. This increase was stronger in patients with neovascular AMD than in patients with PCV and healthy controls, but we did not find statistical significant differences when distributions of differentiation subsets were compared. Interestingly, Ezzat et al. studied human choroid from donor eyes using immunohistochemistry and found that CD8^+^ cells were abundantly present in maculae with AMD [40]. The picture is less clear for PCV. In surgically excised specimens of PCV, Lafaut et al. confirmed presence of aneurysmal vessel dilations but also found islands of possible lymphocytic infiltration [41]. Nakashizuka et al. identified immune cells around the abnormal vessels to be foamy-macrophages; however, the authors did not specifically investigate T-cells or lymphocytes immuno-histochemically [42]. Okubo et al. identified occasional extravascular located macrophages posterior to the dilated vessels, but reported no presence of lymphocytes [43].

CD27 and CD28 on T-cells interact with molecules presented by the antigen presenting cells [37]. Proliferation and differentiation leads to loss of CD27 and CD28: naïve T-cells are CD27^++^ and CD28^++^, CM are CD27^++^ and CD28^++^, EM are CD27^+/−^ and CD28^+/−^, and EMRA are CD27^−^ and CD28^−^ [37]. These changes differ slightly between CD4^+^ and CD8^+^ T-cell populations: in CD4^+^ T-cells, proliferation and differentiation leads first to CD27^−^ and then CD28^−^ later whereas the opposite is the case in CD8^+^ T-cells with first CD28^−^ and then CD27^−^ [31]. These characteristics are in line with the findings in this study (Table 2). An age-related increase in CD27^−^ and CD28^−^ T-cells are well-characterized in human aging [37]. Accelerated loss of CD28 expression, and to some degree also loss of CD27 expression, have also been linked to stimulation with the common cytokine gamma-chain family (interleukin(IL)-2, IL-4, IL-7, IL-9, IL-15, and IL-21) [44,45]. Some of these cytokines have been investigated in patients with PCV and patients with neovascular AMD, in particular IL-4 is found to be increased in both PCV and neovascular AMD [46]. Despite these previous findings, we only found that patients with neovascular AMD, and not patients with PCV, had a significantly more CD27^−^ and CD28^−^ and exclusively as CD8^+^CD28^−^CD27^−^ T-cells. This does not exclude a possible role for IL-4 in PCV, but our results suggest that CD28^−^CD27^−^ T-cells have less significant role in PCV than in neovascular AMD. Our data is the first to suggest that CD8^+^CD28^−^CD27^−^ T-cells may play a role in neovascular AMD. CD8^+^CD28^−^CD27^−^ T-cells are terminally differentiated, have limited capacity of proliferation and survival due to shortened telomeres, and are highly cytotoxic [44]. Interestingly, CD8^+^CD28^−^ T-cells are suspected to reflect exhaustion of the adaptive immune system and it is hypothesized that their accumulation with age contribute to age-related diseases by counteracting the impact of protective T-cells [44]. An acceleration of this process is seen in individuals with cytomegalovirus infection [47,48], which is particularly relevant for AMD since prior cytomegalovirus infection measured as specific IgG titer is demonstrated to be significantly associated with later development of neovascular AMD [49].

CD56^+^ T-cells are an interesting subtype with cytokine-induced cytolytic abilities similar to natural killer cells, but without the natural killer cell-associated receptors of activation or inhibition [50]. Through increased p16 expression, which directly inhibits cellular mitosis, CD56^+^ T-cells have limited ability of proliferation [33]. Proportion of CD56^+^ T-cells increase with age and its expression has been suggested as a marker of immunological aging [33]. CD56^+^ in CD4^+^ T-cells are generally low and its expression is usually much more abundant in CD8^+^ T-cells [33,50]. Exact function of CD56 on T-cells and its possible ligands remains incompletely characterized, but one study identified fibroblast growth factor receptor 1 (FGFR-1) as a ligand for CD56 [51]. FGFR-1 is expressed in human macular specimens of neovascular AMD and experimental studies find increased FGFR-1 expression upon retinal injury from the photoreceptors and the RPE, and FGFR-1 expression itself enhances the expression of VEGF [52,53]. In mice, Oladipupo et al. found that endothelial FGFR-1 is essential for neovascularization following retinal injury [54]. Lemster et al. mapped the production of cytokines in CD56^+^ T-cells upon in vitro CD56 ligation using CD56-specific antibodies [33] and found secretion of interferon gamma (IFN-γ), IL-2, IL-8, IL-13, monocyte chemoattractant protein-1 (MCP-1), macrophage inflammatory protein 1 beta (MIP-1β), and tumor necrosis factor alpha (TNF-α). These circum-stances are interesting in light of the striking differences in the proportion of T-cells that are CD8^+^CD56^+^: patients with PCV did not differ from healthy control individuals, but patients with neovascular AMD had significantly higher proportion of CD8^+^CD56^+^ T-cells. Higher proportion of CD56^+^ T-cells in patients with neovascular AMD were not seen as unspecific general increase in the proportion of CD56^+^ cells, but specifically in the CD8^+^ CM and EM populations which are highly cytotoxic. Considering that neovascular AMD, unlike PCV, is preceded by development of drusen, retinal injury, and RPE dysfunction, an accelerated immunological aging that leads to increased CD56^+^ T-cells may function synergistically to recruit systemic monocytes, activate macrophages, and shape a predominantly pro-inflammatory environment that propel a residential angiogenic drive, expression of VEGF, and consequently development of neovascular AMD. Supporting these hypotheses are the facts that both increased CD56 expression on T-cells and CD8+ memory-to-naïve T-cell ratio have been linked to accelerated aging [31–33,36,38,39], as well as obser-vational studies in patients with AMD and experimental studies in mice of laser-induced CNV, where data suggest significant contribution of systemic and local concentrations of IFN-γ, IL-8, IL-13, MCP-1, MIP-1β, and TNF-α [55]. The link between increased CD56^+^ T-cells and neovascular AMD is independent of important risk factors for neovascular AMD that likely could influence our results, such as cytomegalovirus sero-positivity and smoking as investigated by our group in a previous study [11].

We made another very interesting observation in proportion of CD56^+^ CD8+ T-cells by investigating age-related changes: the significantly higher CD56^+^ was exclusive to the younger group of patients with neovascular AMD (P = 0.00016). Approximately 30 % of individuals above the age of 60 have drusen [56], but only approx. 5 % in 5 years and 15 % in 15 years develop later stages of the disease [3]. Many individuals with early AMD never develop late AMD, but age is the single greatest risk of developing late AMD including neovascular AMD [1]. Hence, younger (≤ 70 years) patients with neovascular AMD constitute an etiolo-gically interesting group. Differences in immunological aging may explain why some relatively young patients with AMD progress to the neovascular subtype of AMD. Results of this study suggest that immunological focus is warranted on young patients with neovascular AMD.

It is important to acknowledge limitations of our study design, which enable correlations with disease but not infer on causality. In addition, we are unable to distinguish whether observed changes preceded development of disease or reflect a systemic adaptation of retinal injury. Possible causation should be invest-tigated in future studies using experimental methods.

In conclusion, we find differences between patients with PCV and patients with neovascular AMD in CD8^+^ T-cells, particularly in the proportion of CD28^−^CD27^−^ cells and CD56^+^ cells. Our data suggest that PCV is similar to healthy controls in terms of T-cell differentiation and aging, and that neovascular AMD is a disease wherein these aspects may be accelerated. Considering that aging is the strongest predictor of neovascular AMD and the findings in this study revealing that younger patients with neovascular AMD have higher CD56^+^ in CD8^+^ T-cells, we propose that younger patients with neovascular AMD may constitute an interesting subgroup that warrants further investigation.

## METHODS

This study was approved by the Regional Committee of Ethics in Research of the Region of Zealand (SJ-379) and followed the principles of ethics in human research as stated in the Declaration of Helsinki. We explained the nature of the study to all participants and obtained oral and written informed consent prior to participation.

### Study design

We recruited patients from the Department of Ophthalmology at Zealand University Hospital Roskilde for this prospective case-control study. Participants were either patients with PCV, patients with neovascular AMD, or age-matched (aged ≥ 60 years) healthy controls. Healthy controls were recruited from healthy biologically unrelated relatives of recruited patients (e.g. husband or wife) to better match the control group in terms of environmental exposures. A direct power calculation for this study was not possible since very few studies have investigated systemic components of PCV and no studies of patients with PCV have described their T-cell differentiation profile or expression of aging marker CD56. However, we have previously investigated CD28^−^CD56^+^ CD8^+^ T-cells in patients with neovascular AMD [11], and based on these experiences we approximated that at least 16 participants would be necessary to study CD56 in CD8^+^ T-cells (assuming an alpha level of 5 %, power level of 80%, means of ~18 % and ~28 % in controls and patients respectively, and a standard deviation of ~10 %). To ensure sufficient power in this study of patients with PCV, we recruited at least 20 in each group and stopped further recruitment after reaching a total of 100 participants.

### Retinal diagnosis

All participants underwent comprehensive ocular examination using slit-lamp bio-microscopy, digital fundus photography, and Spectral Domain Optical Coherence Tomography (SD-OCT). Best-corrected visual acuity (BCVA) was measured in each eye using the Early Treatment of Diabetic Retinopathy Study (ETDRS) chart [57]. Using the Clinical Age-Related Maculopathy Staging (CARMS) definition [58], healthy controls were defined as having less than 10 small drusen (<63 μm) in both eyes with no sign of pigment abnormalities. Fluorescein and indocyanine green angiography (ICGA) was used for macular diagnostics to classify the disease into neovascular AMD or PCV [13]. Neovascular AMD was diagnosed in cases with fibrovascular RPE detachments and choroidal neovascular membranes with subretinal or sub-RPE hemorrhages or fibrosis [35], in addition to the presence of drusen. PCV was diagnosed in cases with one or more polyps seen in the early-phase ICGA with a hypofluorescent halo and with or without BVN [12–14].

### Inclusion and exclusion criteria of participants

We included participants with either PCV in one or both eyes, neovascular AMD in one or both eyes, or healthy retinas. Participants were only included if they had no history of an ongoing immune disease (e.g. any diagnose of cancer, autoimmune diseases, or infectious diseases) or immune modulating treatment (i.e. chemotherapy, immune therapy, steroids, or any other therapy with the purpose of modulating immune function) to avoid blurring of results. Participants were only included if they had not received VEGF inhibitors within 4 weeks (Ranibizumab, Novartis, Basel, Switzerland) or 8 weeks (Aflibercept, Bayer, Lever-kusen, Germany) to avoid potential interaction of systemic antibodies in flow cytometric preparations. Detailed diagnosis with retinal angiography was made on treatment-naïve eyes, but patients were not recruited on their initial visit because recent onset of CNV is associated with acute immune activity [6]. We measured plasma C-reactive protein and post-hoc excluded any participant with levels >15 mg·L^−1^ to avoid including participants with an ongoing immunological acute response [59].

### Clinical data

All participants were interviewed for co-morbidities and treatments, and we crosschecked the data with the patient's electronic patient record. Smoking was noted as current, previous (>100 cigarettes in total, but ceased smoking >12 months), or never smokers. Alcohol use was noted in units/week (one Danish unit = 15 mL or 12 g pure ethanol). General state of physical activity/inactivity was noted using a single question for epidemiological studies [60,61]. Body mass index (BMI) was calculated using height and weight.

### Blood sampling and preparation for flow cytometry

We sampled venous blood in one tube coated with lithium heparin to measure C-reactive protein and one tube coated with ethylenediaminetetraacetic acid (EDTA). The EDTA stabilized blood was prepared for flow cytometric analysis within 4 hours of blood sampling. We obtained a differential count using the automated hematology analyzed Sysmex KX-21N^TM^ (Sysmex Corporation, Kobe, Japan) and the white blood cell count was used to calculate blood volume needed to obtain a constant final number leukocytes in the test tubes (5·10^5^ leukocytes). Sampled blood underwent erythrocyte lysis using red blood cell lysis buffer (Nordic Biosite AB, Täby, Sweden) in dark at room temperature for 10 minutes. We then washed cells three times by centrifuging (5 minutes at 500 G) and re-suspending in an isotonic buffer solution (BD FACSFlow^TM^, BD Biosciences, Franklin Lakes, NJ, USA). We incubated the cells in dark at room temperature for 20 minutes after adding target-specific monoclonal fluorescent antibodies to the test tube (CD4 (Peridinin-chlorophyll protein complex, IgG2a, FAB3791C-100, R&D Systems Inc., Minneapolis, MN, USA), CD8 (Phycoerythrin-CY7, IgG1, 300914, BioLegend, San Diego, CA, USA), CD45ra (Pacific Blue, IgG2b, 304123, BioLegend), CD45ro (Fluorescein Isothiocyanate, IgG2a, MCA461, Bio-Rad Laboratories Inc., Hercules, CA, USA), CCR7 (Brilliant Violet 510, IgG2a, 353232, BioLegend), CD27 (Phycoerythrin, IgG1, 356406, BioLegend), CD28 (Allophycocyanine, IgG1, 301912, BioLegend), and CD56 (Allophycocyanine-CY7, IgG1, 318332, BioLegend)) and flourochrome-matched negative isotypes to a separate tube (Peridinin-chlorophyll protein complex, IgG2a (400250, BioLegend); Phycoerythrin-CY7, IgG1 (400126, BioLegend); Pacific Blue, IgG2b (400331, BioLegend); Fluorescein Isothiocyanate, IgG2a (MCA461, Bio-Rad Laboratories Inc.); Brilliant Violet 510, IgG2a (400172, BioLegend); Phycoerythrin, IgG1 (555749, BD Biosciences); Allophycocyanine, IgG1 (400120, BioLegend); Allophycocyanine-CY7, IgG1 (400128, BioLegend)). After incubation, cells were washed and analyzed using flow cytometry (BD FACSCanto II^TM^, BD Biosciences, Franklin Lakes, NJ, USA) with a sample size of 100.000 singlet leukocytes using a pre-set gate.

### Analysis of flow cytometric data

All flow cytometric data were analyzed using Kaluza Analysis software (Kaluza Analysis v. 1.5.20365.16139, Beckman Coulter Inc., Pasadena, CA, USA) by two independent evaluators (authors Y.S. and M.K.N.) in a blinded fashion to the participants' condition. We obtained T-cell specific results by using following gating strategy: 1) singlet leukocytes (using forward scatter area vs. height), 2) lymphocytes (using forward scatter area vs. side scatter area), 3) CD4^+^CD8^−^ (CD4^+^ T-cells) or CD4^−^CD8^+^ (CD8^+^ T-cells) (using the fluorescence of target-specific antibodies for CD4 vs. CD8). Identification of T-cells are illustrated in Figure 1. Percentages of CD4^+^ and CD8^+^ T-cells in the lymphocyte population were combined with differential count numbers obtained from the automated hematology analyzer to obtain CD4^+^ and CD8^+^ T-cell counts in cells/mm^3^. Functional differentiation profile was defined as naïve T-cells CD45ra^+^CD45ro^−^CCR7^+^, central memory T-cells CD45ra^−^CD45ro^+^CCR7^+^ (CM), effector memory T-cells CD45ra^−^CD45ro^+^ [62]. The distribution of these subsets were investigated in CD4^+^ and CD8^+^ T-cells separately. In CD4^+^ T-cells, we determined % CD27^−^, % CD28^−^, and % CD28^−^ in CD27^−^ cells. In CD8^+^ T-cells, we determined % CD28^−^, % CD27^−^, and % CD27^−^ in CD28^−^ cells. We determined expression of CD56 as % positive in CD4^+^ and CD8^+^ T-cells and in their differentiation subsets using logical Boolean sequences in Kaluza Analysis software. When positive cell populations were not clearly distin-guishable from negative cell populations due to non-specific signaling, the non-specific signaling were eliminated by gating the corresponding negative isotype controls with a threshold of 1 %.

### Statistical analyses

Statistics were performed using SPSS 23 (IBM Corporation, Armonk, NY, USA). Normally distributed continuous data were presented in mean and standard deviation (SD) and compared using parametric tests; otherwise data were presented in median and inter-quartile range (IQR) and compared using non-parametric tests. Categorical variables were presented in numbers and percentages and tested using χ^2^ test or we used the Fisher's Exact test when dealing with small categories (n<5). Correlations with age were made using Spearman's ranked correlation due to lack of normal distribution. When dealing with proportion variables where data were in the extremes (very low or very high), we avoided correlations as such analyses would be extremely prone to outliers and yield unstable results. Figures were made with Prism 7 software (GraphPad, La Jolla, CA, USA). P-values below 0.05 were interpret as sign of statistical significance.
